# Kansas Provider Report of Adolescent Vaccinations in Their Practice

**Published:** 2017-11-30

**Authors:** Serina Padilla, Gretchen Homan, Matt Engel, Carolyn R Ahlers-Schmidt, Kari Harris

**Affiliations:** University of Kansas School of Medicine-Wichita, Department of Pediatrics

**Keywords:** vaccination, immunization, adolescent, survey, vaccination refusal

## Abstract

**Background:**

Kansas falls consistently below average for adolescent vaccination of meningococcal (MCV), human papillomavirus (HPV), and influenza.

**Methods:**

For this study, the members of Kansas Chapter of the American Academy of Pediatrics were emailed a confidential electronic survey soliciting their impressions of vaccination in their practice.

**Results:**

Of 137 providers emailed, 61 (45%) completed the survey. Thirteen providers were excluded as they did not see/vaccinate adolescents or did not complete the survey. Tetanus, diphtheria, pertussis (Tdap), and MCV vaccines were most commonly up to date with 31 (65%) and 20 (42%) respondents reporting greater than 90% immunization rates, respectively. HPV (n = 42, 89%) and influenza (n = 40, 83%) vaccines had refusal rates greater than 25% in most clinics. Most practices (n = 44, 92%) used internal electronic medical records to track vaccinations, although 29 practices (60%) utilized the state immunization information system. Providers requested vaccine-specific patient education tools, positive media coverage, staffing support, and best-practices workshops to support vaccination efforts.

**Conclusion:**

Kansas providers may not be optimizing available resources to enhance these rates, such as Web IZ tracking and immunization reminders. Patient education supplies, specific to HPV and Influenza vaccination, potentially could increase vaccination rates.

## Introduction

Vaccines are readily available in the United States and provide an opportunity to prevent morbidity and mortality. Recommendations for adolescents by the Centers for Disease Control and Prevention (CDC) include tetanus, diphtheria, and pertussis (Tdap) vaccine at age 11 – 12 years, three doses of human papillomavirus (HPV) vaccine starting at age 11 – 12 years, meningococcal conjugate vaccine (MCV) at age 11 – 12 years with a booster at age 16 years, and influenza vaccine annually.^1^ Of these recommended vaccinations, only Tdap is required by the public school system in Kansas.^2^ MCV often is required prior to college attendance. Required vaccination for school attendance is effective in increasing vaccine coverage.^3^,^4^ In part, because of the lack of requirement for HPV vaccine in Kansas schools, there is the potential for state rates to be less than that for other required vaccines.

Healthy People 2020 goals include increasing routine vaccination coverage in adolescents (ages 13 – 15 years), with targets set for each vaccine of 80%.^5^ In 2015, no state met the target coverage for the HPV vaccine series.^6^ HPV is the most common sexually transmitted infection and young people aged 15 – 24 years account for half of new infections each year. The HPV vaccine protects against the most virulent strains.^7^ In the most recent data, 47 states met the Healthy People 2020 target for Tdap vaccine and 36 for MCV, while Kansas had the third lowest rate in the nation for HPV vaccination initiation for girls and was the fifth lowest-ranking state for MCV vaccination.^6^

Providers are key players in vaccine uptake by patients. Provider encouragement is one of the key reasons parents choose to vaccinate their children.^8^,^9^ Likewise, provider hesitancy in vaccine safety or efficacy discourages uptake of vaccines by patients.^8^ Vaccination rates vary widely across Kansas with urban centers typically having higher rates.^10^ Clinical practices performing well in vaccination may have successful policies and protocols in place, but these may not be shared between practices. Methods shown to be effective in increasing adolescent vaccine uptake include patient reminder and recall systems, provider reminders, standing order sets, and immunization information systems (IIS; formally called vaccine registries).^8^,^11^

States are incentivized through Medicaid payment models to have an IIS; WebIZ is Kansas’ IIS. The goal of IISs is to serve as a portable, complete record for the patient, which is especially important if vaccines have been received in multiple locations.^12^ State IISs protect patients and enhance health and safety by supporting communication between immunization providers. IISs can integrate with the provider’s electronic health record software, minimizing potential data entry errors, use information from the CDC to build decision support features, and stay current with updates from that agency.^12^ Reminder/recall systems also have been shown to increase overall vaccination rates for adolescents.^7^,^13^ These systems may originate from the particular electronic health record system or from the state IIS. Further, adolescents, including those from underserved and ethnic minority groups, are receptive to the idea of receiving health information via text messaging and are interested specifically in immunization reminders.^14^,^15^

Despite these strategies, barriers to effective vaccination for adolescents remain. This study aims to evaluate vaccination practices in Kansas, determine barriers to vaccination, and identify tools providers perceive would be beneficial to increase vaccination rates in adolescents.

## Methods

This was a mixed-methods cross-sectional evaluation of provider’s understanding of adolescent vaccination in their practice. A survey was developed with input from pediatricians and researchers. The survey asked about practice characteristics, provider’s interpretation of vaccination practices and coverage, and what resources might improve vaccination coverage.

The Kansas Chapter of the American Academy of Pediatrics (KAAP) provided a list of email addresses for their members. SurveyMonkey_®_ (www.surveymonkey.com) was used to administer the survey which was emailed to all 440 providers on the list with an introductory letter explaining the study. An email reminder was sent after seven days to all providers who had not replied. A final reminder was emailed on day 14, and the survey was closed 21 days after initial contact. The survey consisted of 27 items for vaccinators and 13 items for those who identified they did not vaccinate and was estimated to take five minutes to complete. No personal data were collected with the survey responses.

Providers were excluded from the study if they did not see adolescent patients or if greater than 50% of questions were left blank. Responses were summarized using percentages. Open-ended responses were evaluated for common themes using qualitative content analysis; relevant quotes were extracted from open-ended responses. The study was approved by the University of Kansas School of Medicine-Wichita Human Subjects Committee.

## Results

In total, 440 emails were sent and 137 were opened. Of these, 61 responded to the survey. Five providers (8%) were excluded from analysis because they stated they did not see adolescent patients. An additional five providers (8%) were excluded as they reported not vaccinating adolescents, often relying on another community resource, such as the Health Department, to supply vaccines. Additionally, three incomplete surveys were dropped. Of those included in the analysis (n = 48), 45 providers (94%) worked in general pediatrics and 35 (73%) had been practicing more than 10 years (Table 1).

### Vaccine coverage and refusal rates

**Figure 1:**
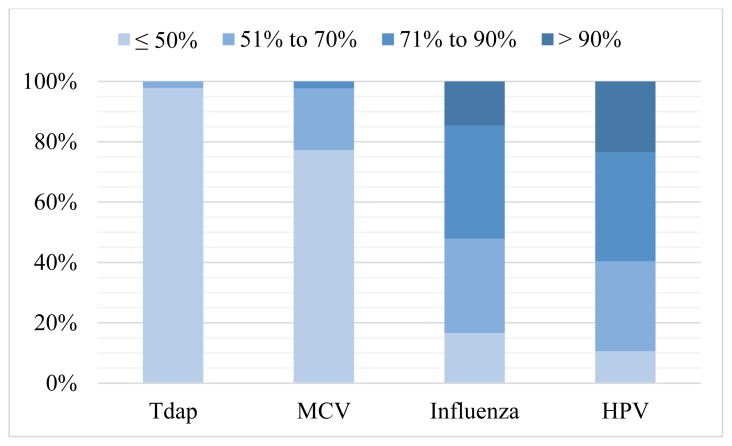
Provider reported vaccination rates. [Tdap: tetanus, diphtheria and pertussis; MCV: meningococcal conjugate vaccine; HPV: human papillomavirus]

Eleven providers (24%) reported at least 80% of their adolescent patients were up-to-date with all routine vaccines (Tdap, MCV, and HPV). Individually, Tdap and MCV vaccines more commonly were reported as up-to-date with 31 (65%) and 20 (42%) respondents reporting greater than 90% vaccination rates, respectively (Figure 1). Influenza and HPV vaccines were given less frequently with only four (8%) respondents and one (2%) respondent reporting vaccinating more than 90% of their patients, respectively. One provider commented, *“…parents look more to what the school districts require than what is recommended by the CDC and AAP”*.

**Figure 2:**
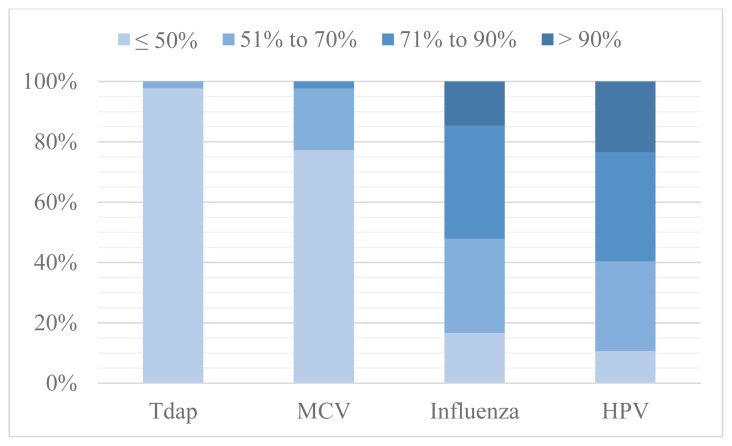
Provider reported vaccine refusal rates. [Tdap: tetanus, diphtheria and pertussis; MCV: meningococcal conjugate vaccine; HPV: human papillomavirus]

Estimated vaccine refusal rates appeared inversely related to vaccination rates, with Tdap and MCV being the least refused (less than 10% of providers reporting) and HPV being reported as refused most frequently (Figure 2). HPV and influenza vaccines had refusal rates greater than 25% in 42 (89%) and 40 (83%) clinics, respectively. Providers indicated in comments that they attempted to supply the recommended vaccinations, but often were met with resistance.

- “*We recommend HPV vaccines at all visits where the child meets age requirements and influenza vaccine during appropriate influenza season (even give influenza vaccine through June). We continue to be surprised at the numbers who refuse HPV and influenza.”*
- “*While resistance to HPV is declining the largest barrier is getting the 3 doses in.”*
- “*The main negative is public perception about vaccine safety. It is slowly changing, but takes regular person-to-person communication to change the perception.”*

### Vaccination practices

All sites reported administering both Tdap and HPV. Two providers (4%) reported not administering MCV and one (2%) reported not administering influenza vaccine. Nurses most frequently administered vaccines (n = 43, 90%), physicians and mid-level providers most frequently counseled and ordered vaccines. Average time estimated counseling on vaccines was six minutes.

While 29 providers (60%) reported that they utilized WebIZ, most (n = 44, 92%) relied on their own internal electronic medical record to track vaccinations. As for reminder systems, 50% of practices (n = 24) relied on phone reminders and nearly 30% (n = 14) did not use any listed method (i.e., phone call, text message, email, mailing, service provided by vaccine manufacturer, making appointment for next vaccine, or patient portal) to remind patients when vaccines were due. One provider reported that reminders were “rarely” used, and another stated that they were working on systems as they developed their new electronic medical record.

Providers were asked whether their practice allowed vaccines to be given to 16-year-old patients without a parent’s consent; 25 practices (53%) said yes for all vaccines whereas two said yes for influenza only. Further, four providers (8%) stated their belief that minors under age 18 could not consent to vaccinations. Eight providers (17%) stated that they would first try to call the parent to get their consent, with one provider clarifying that, if they were unable to get parental consent, they would provide the vaccine without it.

All but one practice regularly provided vaccine information sheets for patients. To ensure information sheets were up-to-date, most providers (n = 27, 56%) reported the clinic waited for emailed alerts that information sheets had been updated. Three providers (6%) reported that a member of their staff (nurse or vaccine coordinator) was responsible for keeping information sheets up to date.

Thirty of the 48 practices (63%) reported that they had worked on a quality improvement project in the past five years related to vaccinations. These data were found to be unassociated with their reported vaccine coverage (Fisher’s exact p > 0.2 for each).

Practices most often requested vaccine-specific patient education supplies, staffing support, and best-practices workshops to support vaccination efforts. Provider continuing education was least requested (n = 4, 8%). In open comments, seven providers (15%) requested efforts to change the parental perception of adolescent vaccination, specifically through conventional articles and social media that support comprehensive vaccination.

“The real help needed is a way to deal with internet and public news that is negative.”

## Discussion

This study showed multiple barriers in our state affect adolescent vaccination uptake. Among these are lack of strong school vaccination requirements, the need for further patient and provider education on HPV vaccination specifically, and underutilization of WebIZ and its resources by practices. Consistent with previous literature, providers in our study reported HPV vaccination rates among the lowest of all adolescent vaccines.^6^,^16^ Currently, Tdap vaccination is the only required vaccination for adolescent entry into Kansas public schools.^2^ In addition, many local colleges require MCV vaccination for entering students. Vaccinations which are not necessary for school attendance (HPV and influenza) have the lowest perceived rate of administration in Kansas. Other barriers to vaccination uncovered in this study included the lack of utilization of the state’s IIS by providers and poor uptake of vaccine reminder systems. Considering that almost a third of those surveyed did not utilize any reminder/recall system, the integrated reminder and recall system that WebIZ offers to its users could be beneficial for many providers.

Providers reported that on average they spent six minutes counseling patients. The estimation may be erroneously large depending on their interpretation of this question and response bias. When vaccines are offered and accepted there are only a few seconds of discussion. Indeed, some providers may not feel that this constitutes counseling, per se. However, providers may recall more readily instances where there is hesitancy, in which case, the process could take several minutes to identify specific questions and provide reassurance to parents.

To support vaccine efforts, providers requested staffing support, best-practices workshops, and patient educational supplies. In particular, patient education specific to HPV vaccination was requested. Vaccine-specific patient education handouts and materials that are up to date and from trusted sources support the conversation between provider and patient.^17^,^18^ Patient education was identified as the area where most support was desired, in particular pertaining to HPV vaccination.

Providers reported frequently that their office and nursing staff were used frequently in the tracking, referral, and promotion of immunizations. As previously recommended,^19^–^21^ continued training of support personnel is vital to successful immunization practices. Patients encounter the office staff first, last, and more often than the provider in most instances. Receiving the same message from the staff and the provider helps to normalize the vaccine and reiterate its value and importance. Conversely, if support staff devalues a vaccine in any way, it negatively affects the patient’s attitudes about vaccination and lessens the likelihood of the provider being successful in advocating for vaccine uptake. Promoting the ability of the staff to use standing orders and feel confident in their abilities can come from best practices workshops.

In addition to educating patients on the importance of vaccinations, provider support measures are needed. The WebIZ system (https://kanphix.kdhe.state.ks.us) has built-in decision support technology that indicates when a vaccine is next due. The registry can generate documents for informed consent to vaccination and can serve as official health documentation that can be used by the patient when accessing social services or for school records. Continuing medical education on reminder and recall systems, including those offered through WebIZ, could increase vaccination rates in Kansas. In addition to assisting practices providing immunizations, steps should be taken to reduce barriers for practices that do not provide immunizations. Practices which do not provide immunizations on site may represent a potential barrier as this could be interpreted by parents as a lack of support by the provider for the immunization.

This study has several limitations. A small percent of providers opened the contact email for the survey. Of those, only 45% completed the survey which could lead to response bias. HPV rates were not collected separately for male and female patients, so the estimated rate of vaccination may be skewed (if respondents averaged both populations) or erroneously high (if respondents only reported female vaccination). In addition, the low utilization of the state vaccine registry by practices makes actual vaccination rates difficult to obtain for our state. Our data showed opportunity for improvement in uptake of these important vaccines. Finally, it is important to understand that barriers in a state with large rural communities may be different than in other regions across the country. Examination of current vaccine practices and identification of gaps is key to finding a solution that will be practical with components that easily are implemented without a large expansion of resources. When success is achieved in expanding a culture supportive of vaccines across the state, the pediatric patients will benefit and enjoy better health as they enter adulthood.

Future research should aim to understand vaccine refusal better, specifically HPV, and to identify specific tools and training for providers to mitigate parental refusal, particularly with regard to HPV and influenza vaccinations. In addition, processes should be implemented to offer comprehensive adolescent vaccination programs in Kansas. These programs should include parental and medical staff education, and ideally should be supported by health care providers, policy makers, and school systems to achieve increased vaccination rates in adolescents.

## Figures and Tables

**Table 1:** Characteristics of vaccinators.

		n (%)
**Specialty**	General Pediatrics	45 (94%)
	Pediatric Subspecialty	2 (4%)
	Family Medicine	1 (2%)
**Time in practice (outside of training)**	0 to 5 years	7 (15%)
	6 to 10 years	6 (13%)
	More than 10 years	35 (73%)
**Percent of patient panel on Medicaid insurance**	Medicaid not accepted	5 (10%)
	Less than 25%	5 (10%)
	25% to 50%	19 (40%)
	51% to 75%	15 (31%)
	Greater than 75%	4 (8%)
**Size of practice**	Small (<5 providers)	18 (38%)
	Mid-sized (5–10 providers)	11 (23%)
	Large (>10 providers)	18 (38%)
Type of practice*	Private practice	21 (44%)
	Multidisciplinary	13 (27%)
	Academic/University affiliated	12 (25%)
	Rural Health Clinic	3 (6%)
	Federally Qualified Health Center/Safety-net	3 (6%)
	State funded clinic/health department	1 (2%)
	House Call	1 (2%)
	Indian Health Service	1 (2%)
	Hospital-Owned	1 (2%)
	Faith-based	1 (2%)

* Multiple responses allowed.
